# Effects of High and Low-To-Moderate Intensity Exercise During (Neo-) Adjuvant Chemotherapy on Muscle Cells, Cardiorespiratory Fitness, and Muscle Function in Women With Breast Cancer: Protocol for a Randomized Controlled Trial

**DOI:** 10.2196/40811

**Published:** 2022-11-11

**Authors:** Olav Vikmoen, Tor Helge Wiestad, Inger Thormodsen, Karin Nordin, Sveinung Berntsen, Ingrid Demmelmaier, Emelie Strandberg, Truls Raastad

**Affiliations:** 1 Department of Physical Performance Norwegian School of Sport Sciences Oslo Norway; 2 The Cancer Center for Education and Rehabilitation Department of Oncology and Medical Physics Haukeland University Hospital Bergen Norway; 3 Department of Public Health and Caring Sciences Uppsala University Uppsala Sweden; 4 Department of Sport Science and Physical Education University of Agder Kristiansand Norway

**Keywords:** resistance training, endurance training, muscle strength, muscle endurance, anthracyclines, taxanes

## Abstract

**Background:**

(Neo-)adjuvant chemotherapy for breast cancer is effective but has deleterious side effects on muscle tissue, resulting in reduced skeletal muscle mass, muscle function, and cardiorespiratory fitness. Various exercise regimens during cancer treatment have been shown to counteract some of these side effects. However, no study has compared the effect of high-intensity training versus low-to-moderate intensity training on muscle tissue cellular outcomes and physical function in patients with breast cancer during chemotherapy.

**Objective:**

The aim of this substudy within the Physical Training in Cancer (Phys-Can) consortium is to evaluate and compare the effects of high and low-to-moderate intensity exercise on muscle cellular outcomes, muscle function, and cardiorespiratory fitness in women with breast cancer undergoing (neo-)adjuvant chemotherapy. We further aim to investigate if the effects of chemotherapy including taxanes on muscles will be different from those of taxane-free chemotherapy.

**Methods:**

Eighty women recently diagnosed with breast cancer scheduled to start (neo-)adjuvant chemotherapy will be randomized to a combination of strength and endurance training, either at high intensity or at low-to-moderate intensity. Testing of muscle function and cardiorespiratory fitness and collection of muscle biopsies from the vastus lateralis muscle will be performed before the first cycle of chemotherapy (or after 1 week, when not possible) (T0), halfway through chemotherapy (T1), and after completion of chemotherapy (T2). It is estimated that approximately 50% of the participants will be willing to undergo muscle biopsies. To separate the effect of the treatment itself, a usual care group with no supervised training will also be included, and in this group, testing and collection of muscle biopsies will be performed at T0 and T2 only.

**Results:**

This study is funded by Active Against Cancer (Aktiv mot kreft) (May 2013) and the Norwegian Cancer Society (December 2018). Inclusion started in December 2016 and the last participant is expected to be recruited in December 2022. As of June 2022, we enrolled 38 (19 with biopsies) participants to the high-intensity training group, 36 (19 with biopsies) participants to the low-to-moderate intensity training group, and 17 (16 with biopsies) participants to the usual care group. Data analyses will start in fall 2022. The first results are expected to be published in spring 2024.

**Conclusions:**

This study will generate new knowledge about the effects of different training intensities for women with breast cancer during chemotherapy treatment. It will give further insight into how chemotherapy affects the muscle tissue and how physical training at different intensities may counteract the treatment side effects in muscles. The results of this study will inform the development and refinement of exercise programs that are effective and compatible with the multidisciplinary management of breast cancer.

**Trial Registration:**

ClinicalTrials.gov NCT05218876; https://tinyurl.com/ysaj9dhm

**International Registered Report Identifier (IRRID):**

DERR1-10.2196/40811

## Introduction

Breast cancer is the most common type of cancer in women in Europe [1]. Advances in treatment and improved survival rates have led to an increased focus on addressing the persistent adverse effects of treatment, including cancer-related fatigue, reduced physical capacity, weight gain, and reduced quality of life [2]. A common (neo)adjuvant treatment for women with breast cancer is chemotherapy with anthracyclines or taxanes or a combination of both. Observational studies have shown an approximately 10% decrease in maximal oxygen uptake (VO_2max_) during chemotherapy [3]. This is concerning since low VO_2max_ has been associated with higher mortality among patients with breast cancer [4]. The reduced VO_2max_ might be related to the reported cardiotoxic effects of anthracyclines [5], but reductions in VO_2max_ are also reported without any signs of impaired cardiac function [6], suggesting there are other mechanisms contributing to the observed decline. Chemotherapy including anthracyclines has been reported to reduce muscle force-generation capacity and other essential muscle functions in both animal studies [7-9] and in patients with breast cancer [10]. Anthracyclines have also been demonstrated to reduce muscle fiber cross-sectional area (CSA) [8] and mitochondrial function [11] in rodents. These findings are supported by analysis on muscle biopsies from 2 small-scale studies on patients with breast cancer undergoing chemotherapy [12,13], which confirm the deleterious effects of chemotherapy on muscle size, mitochondrial structures, and muscle function. However, it is difficult to differentiate if these negative effects on the muscle are the direct effects of chemotherapy, the cancer itself, or indirectly from the reduced levels of physical activity, which is common in patients with cancer [14]. Regardless of cause, loss of skeletal muscle mass has been associated with reduced physical functioning and increased toxicity, that is, poor tolerance to chemotherapy and thus, worse prognosis [15,16].

Our clinical experience suggests that muscle function is more affected during taxane treatment than during anthracycline treatment. One frequent comment from patients during taxane treatment is the feeling of acidification during light and moderate physical activity. Taxanes have been reported to induce peripheral neuropathies [17], and one of the main mechanisms underlying the observed neuropathy is the deleterious effects on mitochondria [17,18]. A similar negative effect on the mitochondria in the skeletal muscle fits well with the abovementioned comments from patients. However, no studies have investigated if the muscular cellular effects of treatment including taxanes are different from those of treatment without taxanes and if including taxanes in the treatment leads to a different response to an exercise intervention. We will recruit patients receiving treatment with anthracyclines, taxanes, or a combination of both. If a sufficient number of patients is given each of the different treatments, we aim to investigate if the effects of treatment including taxanes on the muscle cells are different from those of taxane-free treatment on the muscle cells.

Strength training improves muscular strength and muscle size [19], and endurance training improves mitochondrial volume and mitochondrial function [20]. Therefore, physical training is a potential effective countermeasure to the chemotherapy-induced impairments in skeletal muscle mass and function. In women with breast cancer, physical training has been shown to reduce the loss of muscle strength and cardiorespiratory fitness commonly observed during treatment [2]. However, the physiological mechanisms underlying this protective effect are largely unknown.

Endurance training has been reported to reduce the cardiotoxic effects of anthracyclines in rodents [21-23]. The possible protective effects of physical training on skeletal muscles during chemotherapy for breast cancer have only been studied in 1 small-scale study. Mijwel and colleagues [12] showed that participating in a training program that combined high-intensity intervals to either strength training or aerobic exercise (moderate intensity) during treatment had beneficial effects on muscle fiber CSA and mitochondrial enzymes in the 2 intervention groups. Furthermore, these beneficial effects showed an inverse correlation with changes in cancer-related fatigue, indicating that the training effect on muscle fiber CSA and mitochondrial enzymes during chemotherapy might reduce cancer-related fatigue [12]. However, there is a need for studies including more participants to verify these findings and to investigate the physiological mechanisms underlying this protective effect. Regular exercise during treatment also seems to have several other beneficial effects on both treatment efficiency and reducing the other side effects of treatment [24,25]. Some of these effects seems to be related to increased production of antitumor myokines in the exercising muscles [26], and this aspect will also be investigated in the analyses of muscle biopsies in this study.

To date, most studies have compared a single exercise intervention to usual care or interventions with no physical activity. High-intensity training is shown to induce larger improvements in VO_2max_ and muscle strength in both healthy individuals and in various patient populations [27-29]. However, it is not known to which extent chemotherapy interferes with normal adaptation to physical training. A recent systematic review and meta-analysis reported that longer sessions and higher weekly volume and duration are associated with more beneficial changes in VO_2max_ after endurance training in various populations with cancer during (neo)adjuvant treatment [30]. However, the effects of different training intensities were unclear.

Data from the main study under the Physical Training in Cancer (Phys-Can) consortium showed that combined strength and endurance training with both low-to-moderate intensity and high intensity was feasible in patients with different types of cancer. Furthermore, high-intensity training led to better effects on muscle strength and VO_2max_ compared to low-to-moderate intensity training [29]. However, muscle biopsies were not included in the main study; therefore, how training with different intensities affect muscular cellular outcomes is not known. Thus, there is a need for more studies examining the effect of high versus low-to-moderate intensity training during treatment and especially those including muscle biopsies.

In summary, the direct effects of chemotherapy on muscle tissue in women treated for breast cancer are mostly unknown and previous studies that have investigated the direct effects of (neo-)adjuvant chemotherapy on muscle tissue and how these effects may interfere with the adaptations to strength and endurance training in women diagnosed with breast cancer have had small sample sizes. Furthermore, no previous study has compared the effects of different training intensities on muscle cells in women with breast cancer during (neo-)adjuvant chemotherapy and it is still uncertain whether high-intensity exercise is feasible in all phases of the treatment. Thus, the aim of this study is to evaluate and compare the effects of high and low-to-moderate intensity exercise on muscle cellular outcomes, muscle function, and cardiorespiratory fitness in women with breast cancer undergoing (neo-)adjuvant chemotherapy. We further aim to investigate if the effects of chemotherapy including taxanes on muscle cells are different from those of taxane-free chemotherapy.

Our hypotheses are as follows.

1. Both high-intensity and low-to-moderate intensity strength and endurance training during (neo-)adjuvant chemotherapy will reduce the negative treatment effects on muscle fiber CSA, mitochondrial function, cellular stress, and thus reduce the negative effects on cardiorespiratory fitness and muscle function compared to usual care. High-intensity training will be superior to low-to-moderate-intensity training in counteracting the negative treatments effects.
2. Both high-intensity and low-to-moderate intensity strength and endurance training during (neo-)adjuvant chemotherapy will increase the muscle and blood levels of potential antitumor myokines compared to usual care.
3. Treatment including taxane administration will have larger negative effects on muscle fiber CSA, mitochondrial function, cellular stress, and thus cardiorespiratory fitness and muscle function compared to taxane-free treatment, regardless of the training intensity.

## Methods

### Ethics Approval

This study has been approved by the Regional Committee for Medical and Health Research Ethics South-East, Norway (2015/2360).

### Study Design

**Figure 1:**
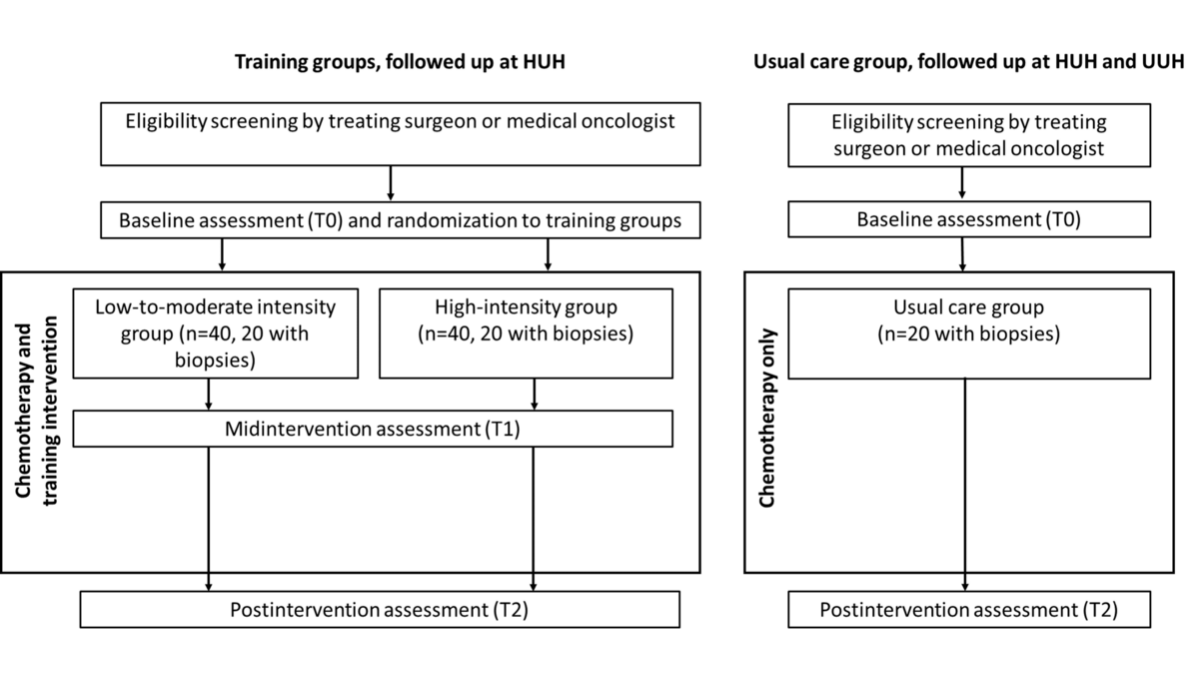
Study flowchart. HUH: Haukeland University Hospital; UUH: Uppsala University Hospital.

This study is a 2-group randomized controlled trial (Figure 1). The participants will be randomly allocated into 1 of the 2 training groups: one group performing the combination of strength and endurance training with high intensity and the other group performing the training with low-to-moderate intensity during (neo-)adjuvant treatment for breast cancer. To measure the effect of the treatment itself, a parallel usual care group receiving no supervised training will be recruited from another pool of patients. This will be from patients living too far away from the study site to participate in training and from a usual care control group from another substudy within the Phys-Can consortium carried out at Uppsala University Hospital [31]. The usual care group will have the same inclusion and exclusion (see below) criteria as the 2 training groups in this study. Muscle biopsies, questionnaires, and blood samples will be collected from the training groups before the first chemotherapy cure (T0). The first 2-4 weeks after cure 1 will be used as a familiarization period for tests and exercises and for completing the remaining T0 tests. Testing will include measurements of physical capacity, body composition, and physical activity levels. All measurements, including muscle biopsies, questionnaires, and blood samples, will be repeated halfway into the treatment (T1) and after completion of treatment (T2). In the participants in the usual care group, measurements will be performed at T0 and T2 only. Training will start between cure 2 and cure 3 and will last throughout the treatment period, which is approximately 6 months.

### Outcomes

The primary outcome for this study is muscle fiber CSA, whereas secondary outcomes include muscle function, cardiorespiratory fitness, regulators of muscle fiber size and function (including mitochondrial enzymes, heat shock proteins, protein control systems, and DNA damage), and myokines with putative antitumor effects. All outcomes are listed in Table 1.

**Table 1:** Outcomes and assessments.

Outcomes and specific variables			Assessment
Muscle fiber size (muscle fiber cross-sectional area)			Cross-sections of muscle biopsies
Number of myonuclei per muscle fiber (myonuclei/fiber)			Cross-sections of muscle biopsies
Number of satellite cells per muscle fiber (satellite cell/fiber)			Cross-sections of muscle biopsies
Proteins involved in muscle hypertrophy (PI3K^a^/Akt^b^/mTOR^c^-pathway, including but not limited to mTOR^c^, P70s6k^d^, 4EBP1^e^, eIF4A^f^)			Western blot
Proteins involved in muscle protein degradation (including but not limited to FOXO^g^, ubiquitin ligase E2, LC3^h^ (I and II), p62^i^, myostatin, as well as ubiquitinated proteins)			Western blot
**Mitochondrial function**			
	CS^j^, COX4^k^, HADH^l^	Western blot	
	Mitochondrial structure	Cross-sections and whole fiber preparations of muscle biopsies	
**Cellular stress**			
	Heat shock protein (Hsp)27, Hsp60, Hsp70	Cross-sections of muscle biopsies, western blot	
	DNA damage	Comet assay	
**Physical function**			
	Muscle strength	1 repetition maximum in chest press and knee extension.	
	Muscular endurance	Repetitions until failure at 30% of 1 repetition maximum	
	Cardiorespiratory fitness	Maximal oxygen uptake	
	Lactate threshold	Blood lactate profile	
Potential antitumor myokines (including, but not limited to interleukin (IL)-6, IL-15, SPARC^m^, TWEAK^n^, IL-8, IL-10, IL-1β, IFN-γ^o^, TNF-α^p^, TNFR1^q^)			mRNA levels by real-time polymerase chain reaction analyses and protein levels by western blot and enzyme-linked immunosorbent assay
**Body composition**			
	Lean body mass, total fat mass	Dual*-*energy X-ray absorptiometry	
	BMI	Weight and height	
Physical activity (level)			SenseWear Armband
Serological outcomes (hemoglobin, creatine, cortisol, high-sensitivity C-reactive protein, total cholesterol, low-density lipoprotein cholesterol, high-density lipoprotein cholesterol, HbA^1c^)			Standard clinical measures
**Quality of life and fatigue**			
	Fatigue	Multidimensional fatigue inventory	
	Pain	Brief pain inventory	
	Health-related quality of life	European Organization for the Research and Treatment of Cancer Quality of Life Questionnaire Core 30, The European Organization for the Research and Treatment of Cancer Quality of Life for breast cancer	
Sociodemographic data (age, partnership, number and age of children living at home, education, income, work and sick leave)			Study-specific questionnaire
Lifestyle data (dietary habits, alcohol consumption, physical activity level, weight, and tobacco use)			Study-specific questionnaire
Behavioral data (motivation, self-efficacy, and barriers to training)			Study-specific questionnaire
Disease-specific information (diagnosis, type, and dose of oncological treatment, adherence to oncological treatment)			Medical records
Adverse events (adverse events occurring during exercise training sessions and during muscle biopsy sampling)			Reported by coaches/technicians

^a^PI3K: phosphoinositol-3-kinase.

^b^Akt: protein kinase B.

^c^mTOR: mechanistic target of rapamycin.

^d^P70s6k: ribosomal protein S6 kinase.

^e^4EBP1: eukaryotic translation initiation factor 4E-binding protein 1.

^f^eIF4A: eukaryotic initiation factor-4A.

^g^FOXO: forkhead box O.

^h^LC3: microtubule-associated protein 1 light chain 3.

^i^p62: ubiquitin-binding protein p62.

^j^CS: citrate synthase.

^k^COX4: cytochrome c oxidase subunit 4.

^l^HADH: 3-hydroxyacyl-CoA-dehydrogenase.

^m^SPARC: secreted protein acidic and rich in cysteine.

^n^TWEAK: TNF-related weak inducer of apoptosis.

^o^IFN-γ: interferon γ.

^p^TNF-α: tumor necrosis factor-α.

^q^TNFR1: tumor necrosis factor receptor 1.

### Participant Recruitment and Eligibility Criteria

Women recently diagnosed with breast cancer starting (neo-)adjuvant chemotherapy (a combination of taxanes and anthracyclines or only one of the treatments or in combination with radiation therapy or endocrine therapy) are recruited from Haukeland University Hospital. Patients in the usual care group (see above) will also be recruited from Uppsala University Hospital. All potential participants must fulfill the following eligibility criteria: (1) diagnosed with stage I-III breast cancer, (2) >18 years old, (3) can understand and communicate in the Norwegian or Swedish language, and (4) scheduled to undergo (neo-)adjuvant chemotherapy with a combination of taxanes and anthracyclines or only one of the treatments or in combination with radiation therapy or endocrines. Women who are (1) not able to perform basic activities of daily living, (2) show cognitive disorders or severe emotional instability, and (3) experiencing other disabling comorbidities that might hamper physical training (eg, heart failure, chronic obstructive pulmonary disease, orthopedic conditions, neurological disorders) will be excluded. All eligible women will receive written information. Women who meet the inclusion criteria will be offered further information and invited to query any question about the study before being invited to participate.

### Sample Size

Power calculations are based on findings in the Physical Exercise and Prostate Cancer trial [32], but findings from the study by Mijwel et al [12] strongly support similar expectations on chemotherapy in patients with breast cancer. With a similar effect on muscle fiber CSA, we need 10 participants in each group to obtain a statistical power of 80% in this study; to further enhance the power up to 95%, we need 16 participants. To account for dropouts during the intervention, we aim to recruit 40 participants to the training groups (20 in each group) willing to undergo muscle biopsies. We expect approximately 50% of the recruited participants will be willing to undergo muscle biopsy; therefore, we aim to recruit 80 participants to the training groups. To increase power on other measurements, the participants unwilling to undergo biopsy will be included in this study. In the usual care group, we also aim to include 20 participants willing to undergo muscle biopsies. This group will consist of patients living too far away from the study site at Haukeland University Hospital and participants in the usual care control group recruited to another substudy within the Phys-Can consortium carried out at Uppsala University Hospital [31].

### Randomization

Participants from Haukeland University Hospital will be randomized in a 1:1 ratio into the 2 training groups stratified by treatment (neoadjuvant or adjuvant treatment). The investigator performing the analyses on muscle biopsies will be blinded for this randomization. As described, participation in the usual care group will be from patients living too far away from the study site at Haukeland University Hospital or from Uppsala University Hospital and will not be randomized.

### Intervention

All participants in the training groups will perform both strength and endurance training throughout the course of treatment with chemotherapy, which is approximately 6 months. Trained coaches will guide both strength and endurance training.

#### Strength Training

The first 2-4 weeks after inclusion will be a familiarization period where the participants become familiar with the exercises and tests as well as how to use the Omni scale for self-reported perceived exertion [33] included in the strength training program. During the familiarization period, there will be a test of 10- and 6-repetition maximum (RM) load, which will provide the participants with individualized training loads. The strength training will be performed as previously described [34]. Briefly, the training consists of 2 supervised sessions per week and include the following exercises: seated leg press, chest press, seated leg curl, seated row, leg extension, and standing overhead press by using dumbbells. The low-to-moderate intensity group will perform 12 repetitions for 3 sets at 50% of 6RM load in the first weekly session and 20 repetitions for 3 sets at 50% of 10RM load in the second weekly session (reporting 5-7 on the Omni scale for perceived exertion) [33]. The high-intensity training group will perform 6 repetitions for 3 sets at 6RM load in the first weekly session and 10 repetitions for 3 sets at 10RM load in the second weekly session (reporting 9-10 on the Omni scale for perceived exertion [33].

#### Endurance Training

During the 2-4 weeks familiarization period, participants will familiarize themselves with the use of the heart rate monitor and perceived exertion by using the Borg scale [35] for monitoring the exercise intensity and perceived exertion. All participants will perform the first session with a coach and receive training on how to use the heart rate monitor. Participants in the high-intensity training group will also be given 1-2 extra session with a coach in a gym. Thereafter, the endurance training is home-based and followed up by a coach and will be performed as previously described [34]. Briefly, the low-to-moderate intensity group perform a continuous-based exercise (running, cycling, walking uphill, or any other endurance-based activity) in bouts of at least 10 minutes at an intensity of 40%-50% of the heart rate reserve. The exercise frequency is recommended to be 2-4 times a week with the main aim to reach 150 minutes of moderate intensity per week. The high-intensity group performs high-intensity interval exercise. The sessions will consist of 2-minute intervals (running, cycling, walking uphill, or any other endurance-based activity) at an intensity of 80%-90% of the heart rate reserve (at the end of the third session) with 2 minutes of rest between intervals. During the first week, after familiarization, each session will consist of 6 intervals. Thereafter, 1 bout will be added every fourth week until 10 bouts per session are reached as the maximum, corresponding to 75 minutes of high-intensity training per week.

### Procedures

#### Muscle Biopsy Sampling

Muscle biopsies are obtained from the midsection of the vastus lateralis muscle under local anesthesia (xylocaine adrenaline, 10 mg·ml^-1^ + 5 μg·ml^-1^, AstraZeneca). Briefly, a 1-2-cm incision will be made in the skin and the fascia of the vastus lateralis muscle. Biopsies are collected using a 6-mm Pelomi needle (Bergström technique) with manual suction to obtain muscle samples (~200 mg). Biopsies will be rinsed in ice cold saline (0.9% NaCl) and carefully dissected free of visual fat, connective tissue, and blood. All pieces but 2 will be frozen in isopentane, precooled on dry ice, and stored at –80 °C for later analysis. The last 2 pieces (~10 mg) will be transferred to 500 μL of RNAlater stabilization solution (Invitrogen) and stored at 4 °C for at least 24 hours before 1 piece is transferred to –20 °C for long-time storage while the RNAlater solution is removed from the last piece before long-term storage at –80 °C.

#### Muscle Analyses

##### Muscle Fiber Size

Muscle fiber CSA represents the primary muscle cellular outcome. Muscle fiber CSA will be measured by immunohistochemical analysis of the cross-sections of the muscle biopsies. Briefly, transverse serial sections of the muscle biopsy (8-μm thick) will be cut using a cryostat microtome at –22 °C and mounted on glass slides. Serial cross-sections will be immunohistochemically stained for fiber types (type I, type IIa, and IIx) for CSA measurements. Muscle fiber CSA will be measured for the different fiber types separately.

##### Regulators of Muscle Fiber Size

The secondary muscle cellular outcomes reflecting the regulators of muscle fiber size are (1) number of myonuclei per muscle fiber, (2) number of satellite cells per muscle fiber, (3) proteins involved in muscle protein degradation (muscle breakdown), and (4) regulators of muscle protein synthesis (local growth factors). Muscle fiber myonuclear and satellite cell content per muscle fiber will be measured by immunohistochemical analysis of the cross-sections of muscle biopsies. Myonuclei and satellite cell contents per muscle fiber will be assessed for the different muscle fiber types separately. Regulators of muscle fiber size, that is, proteins involved in muscle protein synthesis and protein degradation will be measured by western blot analysis in the muscle homogenate. See Table 1 for details.

##### Regulators of Muscle Fiber Function and Cellular Stress

Proteins involved in protection against cellular stress (heat shock protein [Hsp]27, αB-crystallin, Hsp60, and Hsp70) as well as enzymes involved in mitochondrial function (citrate synthase, cytochrome c oxidase subunit 4, and 3-hydroxyacyl-CoA-dehydrogenase) will be assessed in muscle homogenates by western blot analysis. In addition, mitochondrial structures will be studied in cross-sections and whole fiber preparations of muscle biopsies by immunohistochemistry. DNA damage and repair will be assessed using the comet assay [36].

##### Myokines With Potential Antitumor Effects

Exploratory analyses on the effects of the training on the expression levels of myokines, previously proposed to have an antitumor effect, will be conducted. Relevant targets, including, but not limited to, interleukin (IL)-6, IL-15, secreted protein acidic and rich in cysteine, and TNF-related weak inducer of apoptosis will be evaluated at the mRNA level by real-time polymerase chain reaction analyses (RNA extracted from biopsies) and at the protein level by western blot and enzyme-linked immunosorbent assays (muscle and blood samples). Blood samples will be obtained by venipuncture and participants are asked to avoid smoking and alcohol and not to engage in any strenuous physical activity 24 hours before the blood sample collection. The levels of IL-6, IL-8, IL-10, IL-1β, IFN-γ, tumor necrosis factor (TNF)-α, and TNFR1 will be measured using enzyme-linked immunosorbent assay–based methods. Frozen sera will be saved for further analyses that can be included later.

#### Physical Functioning

##### 1RM Testing

1RM testing will be performed as described previously [34] in chest press, leg press, and knee extension. To secure the validity of the 1RM tests, all participants will undertake a familiarization session prior to these assessments.

##### Muscle Endurance

Muscle endurance will be measured as the number of repetitions the patient is able to perform in a continuous set at 30% of 1RM at the corresponding time point in knee extension.

##### Cardiorespiratory Fitness

Cardiorespiratory fitness will be measured as VO_2max_ during maximal walking/running until exhaustion on a treadmill (PPS Med 55, Woodway Inc). The protocols start at 5 km/h with an incline of 5%. The inclination increases with 1% every minute until it reaches 12%, from which the speed increases by 0.5 km/h per minute until exhaustion. Oxygen consumption and minute ventilation will be measured continuously using an oxygen analyzer (Oxycon Pro, Erich Jaeger GmbH; Vyntus CPX, Vyaire Medical GmbH). Heart rate will be measured using a heart rate monitor (T34, Polar Electro KY).

#### Blood Lactate Profile

The patients will walk or run in 5 minutes at bouts with increasing submaximal workloads. Heart rate will be monitored continuously, and capillary blood samples will be taken and analyzed for lactate levels (Lactate Scout+, EKF GmbH) after each workload. The test will terminate when the patients show increased lactate concentrations by more than 1.6 mmol/L from the last workload or when the lactate increases above 4 mmol/L.

#### Body Composition

Total and regional lean body mass and fat mass together with bone mineral density will be measured by dual energy X-ray absorptiometry (iDXA, GE Lunar). Participants will be scanned from head to toe in a supine position, providing values for total and regional lean body mass fat mass, bone mineral content, and bone mineral density.

#### Assessment of Physical Activity Level and Physical Training

Participants’ physical activity level will be measured using SenseWear Armband Mini (BodyMedia Inc). All participants will be instructed to wear the SenseWear Armband for 7 consecutive days. Only valid days with at least 80% wearing time will be included in the analyses. The step count cut points corresponding to moderate intensity will be 3 metabolic equivalents of task [37]. SenseWear Armband data will be analyzed with the SenseWear software (SenseWear Professional Research Software Version 8.1, BodyMedia Inc). Participants will be instructed to keep a logbook of all endurance trainings and strength trainings. In this logbook, the duration and subjective intensity measured with the Borg scale [35] of all endurance training sessions are noted. For the strength training, the load, number of repetitions, number of sets, and the perceived exertion with Omni scale are noted for each session.

#### Quality of Life, Fatigue, and Pain

The European Organization for the Research and Treatment of Cancer Quality of Life Questionnaire Core 30 [38] and diagnosis-specific modules (The European Organization for the Research and Treatment of Cancer Quality of Life for breast cancer) will be used to assess the quality of life. Fatigue will be assessed using the Multidimensional Fatigue Inventory [39], and pain will be assessed using Brief Pain Inventory [40].

#### Background Variables

Participants will provide self-reports about age, partnership, number and age of children living at home, education, income, work, sick leave, dietary habits, alcohol consumption, physical activity level, weight, tobacco use, motivation, self-efficacy, and barriers to training by using a study-specific questionnaire. In addition, past illnesses and other medical problems are recorded. Information about the medical situations such as treatment, stage of disease, and comorbidity as well as chemotherapy treatment compliance and adverse events will be collected at all 3 assessment points (T0, T1, and T2) from medical records.

### Statistical Analyses

Data will be analyzed according to the intention-to-treat principle. Analyses will include standard descriptive statistics, 2-sided *t* tests, correlation, regression, and 2-way repeated-measures analysis of variance or the comparable nonparametric test as necessary to examine the differences between and within groups at T0, T1, and T2. In addition, a per-protocol analysis, that is, adherence to the protocol, will be conducted. Should imbalances in important variables be detected, sensitivity analyses will also be added including these as covariates in the model.

## Results

This study is funded by Active Against Cancer (Aktiv mot kreft) (May 2013) and the Norwegian Cancer Society (December 2018). It has been registered at ClinicalTrials.gov (identifier NCT05218876). At Haukeland University Hospital, inclusion started in December 2016 and the last participant is expected to be recruited in December 2022. As of June 2022, we enrolled 38 (19 with biopsies) participants to the high-intensity training group, 36 (19 with biopsies) participants to the low-to-moderate intensity training group, and 5 (4 with biopsies) participants to the usual care group. The recruitment to the usual care group from Uppsala University Hospital started in December 2018 and is finished with a total of 12 patients completing all data collection. Data analyses of the patients from Haukeland University Hospital will start in fall 2022. Data analyses of the patients at Uppsala University Hospital started in January 2022 and is ongoing. The first results are expected to be published in spring 2024.

## Discussion

The main aim of this study is to compare the effects of a high-intensity strength and endurance training program with those of a low-to-moderate intensity strength and endurance training program on muscle cellular outcomes, muscle function, and cardiorespiratory fitness in women undergoing breast cancer chemotherapy. These results will also be compared with those of the group treated with usual care to investigate how (neo-)adjuvant treatment with chemotherapy will affect these variables and how high and low-to-moderate intensity trainings can counteract the effects of treatment. We hypothesize that the usual care control group will experience negative treatment effects on muscle fiber CSA and mitochondrial function, leading to reduced muscle function and cardiorespiratory fitness. We further expect that both high-intensity training and low-to-moderate intensity training performed by the training groups will counteract the negative treatment effects and that high-intensity training will be superior to low-to-moderate-intensity training. The results of our study are expected to provide insights on how regular exercise during treatment may counteract the side effects of chemotherapy on physical functioning and muscle tissue and how training intensity impacts these effects. Such knowledge can be used to design effective physical exercise programs, helping an increasing number of individuals with breast cancer during and following chemotherapy and possibly reducing the long-lasting side effects and ultimately improve the quality of life.

Forty women recently diagnosed with breast cancer, with 20 in each group, will give us a larger study population than those in previous studies on muscle cellular outcomes [12,41,42] to draw conclusions from. Furthermore, to our knowledge, this will be the first randomized controlled study comparing the effectiveness between high-intensity strength and endurance training and low-to-moderate intensity strength and endurance training during (neo-)adjuvant treatment on muscle cellular outcomes in patients with breast cancer. We are also recruiting participants who are not willing to undergo muscle biopsies, giving us an even larger study population when analyzing the other outcomes. As high-intensity training is shown to induce larger improvements in maximal oxygen uptake and muscle strength in both healthy individuals and in various patient populations [27,28], it should also be more effective in patients with breast cancer during chemotherapy. However, it is not known to which extent chemotherapy interferes with normal adaptation to physical training, and the high-intensity training is severely more challenging. Consequently, high-intensity training may be less feasible during chemotherapy, and lower adherence to the planned training in some periods of treatment may reduce training effectiveness. However, the feasibility of the current high-intensity training program has been confirmed in the large-scale Phys-Can study [29], in which ~75% of the participants completed the 6-month training program.

Although the primary outcome of this study is muscle fiber CSA, we are also including a wide range of biological measurements, including specific proteins involved in skeletal muscle hypertrophy, protein degradation/protein control, and regulators of muscle fiber function. These analyses will provide further insight into the underlying mechanism through which chemotherapy affects muscle tissue and therefore, muscle function, and how training with different training intensities could be used as a therapeutic measure to counteract the side effects of chemotherapy. We will recruit patients undergoing chemotherapy with anthracyclines, taxanes, or a combination of both. Given a large enough number of patients receiving different treatments, this will give us the opportunity to investigate if different chemotherapy regimens affect the adaptations to training at different intensities. Due to individual treatment protocols, there probably will be differences between participants in the treatment regimen, for example, different type and doses of chemotherapy. This might lead to differences between the 2 training groups and between the training groups and the usual care control group in treatment. The lack of randomization to the usual care control group is also a limitation. This will, together with the fact that most participants in this group are treated at a different site than the training groups, further increase the risk of differences between the training groups and the usual care control group in the treatments and other relevant factors. The results from this study are planned to be published in scientific peer review journals and at scientific congresses.

In summary, previous research underlines the positive potential of regular physical exercise during cancer treatment on outcomes such as physical function, mental health, fatigue, and quality of life in women with breast cancer [10,43,44]. However, research on the specific cellular effects of training with different intensities has not been performed. This study will provide important information on the effects of a high-intensity training versus low-to-moderate intensity strength and endurance training programs on skeletal muscle cellular outcomes, muscle function, and cardiorespiratory fitness in women diagnosed with breast cancer undergoing chemotherapy. It will also give important information about the cellular mechanisms through which chemotherapy may reduce physical performance and how training with different intensities may counteract these side effects. This knowledge can be used to design training programs that are both effective and feasible for patients with breast cancer during treatment to counteract the side effects of chemotherapy and ultimately increase the daily function and quality of life.

## Appendix

Multimedia Appendix 1 Peer review report by: Norwegian Cancer Society (Oslo, Norway).

## Abbreviations

CSA: cross-sectional area
Hsp: heat shock protein
IL: interleukin
Phys-Can: Physical Training and Cancer
RM: repetition maximum
T0: test period before the first chemotherapy cure
T1: test period halfway into the treatment
T2: test period after completion of treatment
TNF: tumor necrosis factor
VO_2max_: maximal oxygen uptake

## Data Availability

The data that will be generated from this study is planned to be included in the scientific articles that will be published. Data not included in published articles will be available from the corresponding author on reasonable request.
