# Supplementary material for: Effects of High and Low-To-Moderate Intensity Exercise During (Neo-) Adjuvant Chemotherapy on Muscle Cells, Cardiorespiratory Fitness, and Muscle Function in Women With Breast Cancer: Protocol for a Randomized Controlled Trial
Source: JMIR Res Protoc. 2022 Nov 11;11(11):e40811. doi: 10.2196/40811 (PMC9700233; doi:10.2196/40811)
Supplement: Multimedia Appendix 1 [file resprot_v11i11e40811_app1.pdf]

NORWEGIAN **CANCER** SOCIETY

Date: 23.10.2018

Dear Truls Raastad,

We are pleased to inform you that your grant application to the Norwegian Cancer Society's Open Call 2018 for the research project *Effects of high intensity exercise on muscle cellular outcomes during adjuvant treatment in breast cancer patients* has been approved.

The total project grant for the period 01.01.2019 to 31.12.2021 is NOK 3880000.

Please note that if your project receives only partial funding, the Norwegian Cancer Society may request a revised project proposal.

Your application has been carefully reviewed and considered by the peer review committee specified in your application. Grant applications are ranked on the basis of merit by one of five discipline-specific peer review committees, each of which is composed of six international researchers.

User representatives have taken part in the assessment process in each peer review committee. The aim of user representation is to assess whether user involvement is relevant, and if so, to what degree it is implemented in the project.

An overview of committee 5: Epidemiological, health, and social science research's evaluation of your proposal is provided below:

Relevance to cancer: Ja
Scientific quality: 5
Qualifications of the project manager and project group: 5
Impact: 4
Feasibility: 4

Overall score: 4.67 (considered fundable > 4.5)

The peer review committee´s comment to your proposal is provided below.

This project is part of a larger study, investigating effects of high or low-moderate exercise on outcomes following adjuvant chemotherapy for breast cancer. While study methods are not very well described, the study seems well planned and addresses an important topic. That said, there is a paucity of specific information on study design in the application, limiting the possibility to evaluate it fairly. For example, little or no details are given about details of the study setup, with regards to how patients will be randomized etc. Similarly, although the researchers seem to have prior experience of similar studies, they provide insufficient detail about the power calculations, leaving the reviewers uncertain whether it will really be able to detect meaningful differences.

The awarded grant must be accepted by your project administrator. You will receive a copy of the formal contract between Norges Idrettshøgskole and the Norwegian Cancer Society.

Please note that you may not receive funding from two different sources for overlapping projects. In case of funding from both the Norwegian Cancer Society and another source, funding from the other source should be prioritized. In case of funding of two projects from two separate calls from the Norwegian Cancer Society within the same time period, you may only receive one grant.

General information about this year's call will soon be posted on our website. Please note that the right of appeal is limited to case handling errors and misuse of power, and must be received by the Norwegian Cancer Society within three weeks of receiving this letter. We kindly ask that questions regarding the evaluation process and/or the right to appeal be directed to forskningsadministrasjon@kreftforeningen.no.

As a recipient of research funding, we welcome you to this year's award ceremony. The ceremony will take place on Tuesday October 30 from 12.30-14.30 at the Norwegian Cancer Society's Science Centre, located in Kongens gate 6, Oslo. We would very much like you to be present at this event!

Please register to attend here by Thursday October 25 at 16.00.

Sincerely,
The Norwegian Cancer Society

Anne Lise Ryel
Secretary General

Post address: Postboks 4 Sentrum, 0101 Oslo
Visitation address: Kongens gate 6, 0153 Oslo
Telephone: 21 49 49 21
Email: forskningsadministrasjon@kreftforeningen.no
www.kreftforeningen.no

CC: Kristian Sollesnes
